# Magnetic Nanotrap Particles Preserve the Stability of Venezuelan Equine Encephalitis Virus in Blood for Laboratory Detection

**DOI:** 10.3389/fvets.2019.00509

**Published:** 2020-01-28

**Authors:** Ivan Akhrymuk, Shih-Chao Lin, Mei Sun, Anurag Patnaik, Caitlin Lehman, Louis Altamura, Timothy Minogue, Ben Lepene, Monique L. van Hoek, Kylene Kehn-Hall

**Affiliations:** ^1^National Center for Biodefense and Infectious Diseases, School of Systems Biology, George Mason University, Manassas, VA, United States; ^2^United States Army Medical Research Institute of Infectious Diseases, Fort Detrick, Frederick, MD, United States; ^3^Ceres Nanosciences, Inc., Manassas, VA, United States

**Keywords:** alphavirus, diagnostics, surveillance, Venezuelan equine encephalitis virus, nanotrap particles, nanoparticles

## Abstract

Most of the modern techniques used for identification of viral-induced disease are based on identification of viral antigens and/or nucleic acids in patient's blood. Diagnosis in the field or in remote locations can be challenging and alternatively samples are shipped to diagnostic labs for testing. Shipments must occur under controlled temperature conditions to prevent loss of sample integrity. We have tested the ability of magnetic Nanotrap® (NT) particles to improve stability and detection of Venezuelan equine encephalitis virus (VEEV), viral capsid protein, and viral genomic RNA in whole human blood at elevated temperature and prolonged storage conditions. NT particles have previously been shown to capture and enrich multiple pathogens including respiratory syncytial virus, influenza virus, coronavirus, and Rift Valley fever virus. Our study indicates that samples incubated with NT particles had detectable levels of infectious VEEV in blood equal to or greater than samples without NT treatment across all temperatures. Viral RNA detection was increased in the presence of NT particles at later time points (72 h) and higher temperature (40°C) conditions. Likewise, detection of VEEV capsid protein was enhanced in the presence of NT particles up to 72 h at 40°C. Finally, we intranasally infected C3H mice with TC-83, the live attenuated vaccine strain of VEEV, and demonstrated that NT particles could substantially increase the detection of VEEV capsid in infected blood incubated up to 72 h at 40°C. Samples without NT particles had undetectable capsid protein levels. Taken together, our data demonstrate the ability of NT particles to preserve and enable detection of VEEV in human and mouse blood samples over time and at elevated temperatures.

## Introduction

Venezuelan equine encephalitis virus (VEEV) is one of the most neglected viruses among biowarfare agents. It is classified as a Category B biothreat pathogen by National Institute of Allergy and Infectious Diseases (NIAID), USA, due to its high dissemination rate, minimal infectious dose to induce disease in human, and the requirement for specific and enhanced diagnostic capacities. VEEV causes disease with symptoms ranging from influenza-like illness to more severe illnesses including myalgia, arthralgia, and neurological disorders that can lead to lethal encephalitis in susceptible hosts. All these characteristics made VEEV an attractive candidate for weaponization (1). VEEV was first isolated from an infected horse brain during a VEEV outbreak in Venezuela in 1938 followed by major outbreaks in Venezuela and Columbia in 1960s (2), infecting thousands of people and animals. Despite its high contagiousness, VEEV has drawn little public attention due to only sporadic outbreaks occurring in Central and South America since 1995. Other reasons for being neglected may be a low mortality rate in humans (<1%) while as high as 90% in horses, as well as no reports of VEEV outbreaks in the US since the only epidemic outbreak in Texas in 1971 (3). However, the spillover of VEEV infection from infected horses to humans during epidemics remains a concern.

VEEV is an arthropod-borne epizootic RNA virus, belonging to the *Togaviridae* family, genus *Alphavirus*, and is maintained within lower animals (rodents) and mosquitos (4). The transmission of VEEV is primarily mediated by mosquitoes, where it replicates in salivary glands and is passed to hosts, such as human and horses with overt symptoms (5, 6). Moreover, aerosolized VEEV can be directly disseminated and can infect humans or susceptible experimental animal models, causing encephalitis, and possibly limb paralysis (7–9). Historically, since the late 1930s the former Soviet Union regarded VEEV as an operational biological weapon to incapacitate the rear services and reinforcement behind the front line, leading to the spread of the disease among infected individuals with flu-like symptoms that are difficult to distinguish from epidemic influenza outbreaks. Weaponized VEEV was not expected to kill the soldiers, but cause panic and ultimately maim the military targets (10). Notably, there are no effective antiviral agents available and approved by the US Food and Drug Administration (FDA) and only supportive treatment is available for humans. As a result, it is important to establish a preventive surveillance system based on prompt diagnosis methods.

Viral stability is an essential factor for diagnosis of VEEV to confirm the presence of virions or viral RNA in a clinical sample. The current diagnostic approaches to confirm VEEV infection in humans or horses rely on direct detection of viral nucleic acids in serum or spinal fluid samples during the acute-phase of infection using reverse-transcription PCR (RT-PCR) (11) and ELISA for VEEV-specific IgM (12). However, despite high sensitivity of the above-mentioned methods, false negative results can be obtained if samples have been collected during the initial asymptomatic phase of infection where the viral load is low (13–15). Moreover, the necessity of extra steps of plasma or serum preparation complicates fast virus identification and diagnostic. Therefore, virus stabilization directly in collected sample without extra preparation steps is highly desirable. Ideally, supplementation of the blood collection device with some type of stabilization agent that can minimize pathogen loss during sample transportation and storage at ambient temperatures is appealing. In view of these challenges, Nanotrap® (NT) particles were evaluated for their ability to stabilize VEEV. NT particles are hydrogel polymer particles comprised of N-isopropylacrylamide (NIPAm), allylamine (AA), and crosslinked with N,N′-methylenbisacrylamide (BIS). These particles are functionalized with various dye affinity baits that facilitate capture and retention of analytes from complex biological matrixes (such as blood, saliva, nasal swabs and urine) and concentrate them into a smaller volume (16–20). Previous work with NT particles has shown the benefit of their use in the enrichment of infectious virus and viral genomic material of Rift Valley fever virus (RVFV), coronavirus, influenza virus, and respiratory syncytia virus (19, 21).

In this study, we sought to apply new magnetic NT particles that consist of NIPAm copolymers functionalized with reactive red 120 to evaluate the efficacy of preservation of infectious VEEV, viral RNA, and VEEV capsid protein in whole blood samples at ambient and elevated temperature as well as at low and high humidity conditions. Our results indicate that: (i) magnetic NT particles enhance preservation of infectious VEEV in whole human blood at 40°C; (ii) NT maintain significantly higher levels of VEEV RNA in whole human blood at 40°C; (iii) NT retain their functional activity at both normal and elevated humidity conditions and significantly preserve VEEV infectivity in such an environment; and (iv) blood samples from VEEV TC-83 infected animals are better protected from capsid protein degradation if they are incubated with NT. Our results demonstrate for the first time the capability of NT particles to stabilize virus in blood at elevated temperatures, the direct interaction of NT particles with VEEV (via transmission electron microscopy), and the utility of NT particles with viral clinical samples.

## Materials and Methods

### Viruses, Body Fluids, and NT Particles

VEEV-TC83 viral stocks were produced from electroporation of *in vitro* transcribed viral RNA generated from the pTC83 plasmid [a kind gift from Ilya Frolov, The University of Texas Medical Branch at Galveston] as described (22). All experiments were performed under BSL-2 conditions. Whole human blood and plasma was purchased from BioIVT (www.bioivt.com). All of the NT particles were provided by Ceres Nanoscience, Manassas, VA (ceresnano.com).

### NT Particle Screening

VEEV TC83 was diluted to 1 × 10^6^ PFU/mL in whole human blood followed by incubation with NT particle suspension (0.5 mg of NT/ml of sample or 1.25 mg of NT/ml of sample) or without NT particles, rotating for 30 min at room temperature. Viral RNA was purified using RNeasy kit (Qiagen). The amount of the viral RNA was determined by RT-qPCR using SuperScript™ III Platinum™ SYBR™ Green One-Step qPCR Kit w/ROX (Thermo Fisher Scientific) with the following set of primers (Integrated DNA Technologies): 5′-TCTGACAAGACGTTCCCAATCA-3′ and 5′-GAATAACTTCCCTCCGACCACA-3′. The Taq-Man probe (5′-6-carboxyfluorescein-TGTTGGAAGGGAAGATAAACGGCTACGC-6-carboxy-N,N,N′,N-tetramethylrhodamine-3′) was designed against the RNA packaging signal as described previously (23). RT-qPCR was performed using a StepOne Plus Real Time PCR System instrument from ABI. Fold enrichment was calculated based on the enriched viral genomic copy number divided by those in the “without NT particle” group.

### Virus Enrichment Experiment

VEEV TC83 was diluted to 1 × 10^6^ PFU/mL in whole human blood or plasma followed by incubation with or without NT particles at the indicated humidity and temperature conditions for various time points. Viral RNA was purified using a combination of a TriZol® LS from Ambion and RNeasy mini kit (Qiagen). Briefly, whole human blood containing viral virions was mixed in 1:3 ratio with TriZol LS. Hundred microliter of PBS were added to the sample in order to increase the amount of the aqueous fraction. Samples were vortexed and 200 μL of chloroform were added to the blood TriZol LS mixture. After intensive vortexing and spinning down the upper aqueous fraction containing nucleic acids was collected and used for RNA purification by RNeasy kit (Qiagen) according to the manufacturer's protocol. Purified RNA was used for cDNA synthesis followed by PCR reaction (30 cycles) using a One Step RT-PCR kit (ThermoFisher Scientific). For this purpose, the following set of primers was used: 5′-CTG CTC GCC AAT GTG ACG TTC-3′; 5′-AGC CTG CTC TGT TGA CTA TAG TGT TAT ACG-3′. To visualize the quantity of viral cDNA 10 μl of the PCR product were loaded on 1% agarose gel supplemented with ethidium bromide, followed by gel electrophoresis. Samples were visualized on a ChemiDoc instrument from Bio-Rad and densitometrically analyzed using Image Lab Software from Bio-Rad.

### Plaque Assay

1 × 10^6^ PFU of the VEEV TC-83 were spiked into whole human blood supplemented (1.25 mg of NT/ml of sample) or not supplemented with NT particles. Samples were incubated at the indicated temperature and humidity conditions for the designated amount of time. At the end of the incubation, NT particles containing samples were placed on a magnet rack to separate NT particles from the blood. Particles were resuspended in 100 μl of PBS and used for standard plaque assay as described elsewhere (24). Samples that were incubated without NT particles were processed using the same method.

### Western Blotting

VEEV TC83 was diluted to 1 × 10^6^ PFU/mL in plasma followed by incubation with or without NT particles (1.25 mg of NT/ml of sample) at indicated humidity and temperature conditions for various time points. Blue lysis buffer (2× Novex Tris-Glycine Sample Loading Buffer SDS, T-PER Tissue Protein Extraction Reagent, 0.5 M EDTA, complete protease cocktail tablets, 0.1 M Na_3_VO_4_, 0.1 M NaF, and 1 M DTT) was added to the samples in 1:1 volume ratio, followed by boiling at 95°C for 10 min. Protein lysates were then electrophoresed in a 4–12% of Bis-Tris SDS-PAGE gel and transferred to a PVDF membrane. A 1:2,000 dilution of primary anti-VEEV capsid antibody (Cat#NR-9403, BEI, Manassas, VA USA) in 5% non-fat milk blocking buffer was applied on the membrane at 4°C for overnight incubation followed by washing with PBS + 0.05% Tween 20 three times. Horseradish peroxidase (HRP)-conjugated anti-goat IgG secondary antibody (ThermoFisher Scientific) at a 1:2,000 dilution was incubated with the membrane for 1 h at room temperature. The membrane was washed an additional three times with PBS + 0.05% Tween 20 before adding SuperSignal™ West Femto Maximum Sensitivity Substrate (Thermofisher Scientific) to develop the membrane and image the chemiluminescent signal. Samples were visualized on ChemiDoc instrument from Bio-Rad and densitometrically analyzed by the Image Lab Software from Bio-Rad.

### Animal Experiment

A total of 27 C3H/HeN mice (6–8-week-old) were randomly divided into three groups (*n* = 9) and VEEV-TC83 was diluted to 2 × 10^7^ PFU/mL with PBS followed by intranasally administrated to anesthetized mice in the amount of 20 μL/mouse. By 2, 3, and 4 days post-infection, 9 mice were sacrificed at each time point and the blood was collected in EDTA-treated tubes to prevent coagulation. RT-qPCR was performed to quantify the level of viremia and the 6 samples with the highest viremia were pooled together and then incubated with or without NT particles to compare the capsid stability by western blot analysis. Experiments were performed in animal biosafety level 2 (ABSL-2) laboratories in accordance with the National Research Council's *Guide for the Care and Use of Laboratory Animals* (25) and under George Mason University IACUC protocol number 0384.

### Protein Docking Prediction

The VEEV structural protein (E1, E2, E3, and capsid) was retrieved from PDB website (ID: 3J0C) and the chemical structure of reactive red 120 was downloaded from Chemspider website. The heterodimer of E1 and E2 were extracted from the total protein structure with Molsoft ICM-Browser followed by submitting it to Swissdock for prediction of possible docking pockets (26). The docking models were displayed in ball-and-stick style of reactive red 120 and residue surface style of each amino acid on the heterodimer and represented images were selected based on two of the lowest free energy values (ΔG) on each subunit.

### Negative Staining and Electron Microscopy (EM) Imaging

Inside the laboratory biosafety cabinet (BSC) the NT particle—VEEV suspension was mixed well with the same volume of 4% Glutaraldehyde to achieve a final concentration of 2% Glutaraldehyde. Virus samples were inactivated with 2% Glutaraldehyde inside a BSC for 24 h according to industry standard practice (27), prior to removal and transfer to the BSL-2 EM facility. A drop (8 μl) of the glutaraldehyde treated sample was placed onto a formvar/carbon coated transmission electron microscopy (TEM) grid for 10 min in a moist chamber to reduce evaporation. This step ensures that the grid did not dry. Using fine forceps to hold the grid, the liquid was wicked away from the grid surface from the side with filter paper. The grid was then washed three times by touching the grid to the surface of drops of deionized (dI) water. Remaining water was wicked away by touching filter paper to the side of the grid. A small drop of 1% phosphotungstic acid (PTA) was applied to the grid and allowed to remain from 10 s to 1 min depending on the sample. The stain was wicked away by touching the edge of the grid to a piece of filter paper. The grid was air dried at room temperature and stored for subsequent TEM imaging.

TEM grids were evaluated on a JEOL 1011 transmission electron microscope at 80 kV and all digital images were acquired using an Advanced Microscopy Techniques (AMT) camera system.

### Statistical Analysis

Kruskal-Wallis test and unpaired *t*-test were performed throughout this study to calculate the statistical significance among groups with and without NT particles unless indicated elsewhere.

## Results

### Screening of NT Particle Types for Enriching VEEV-TC83

**Figure 1:**
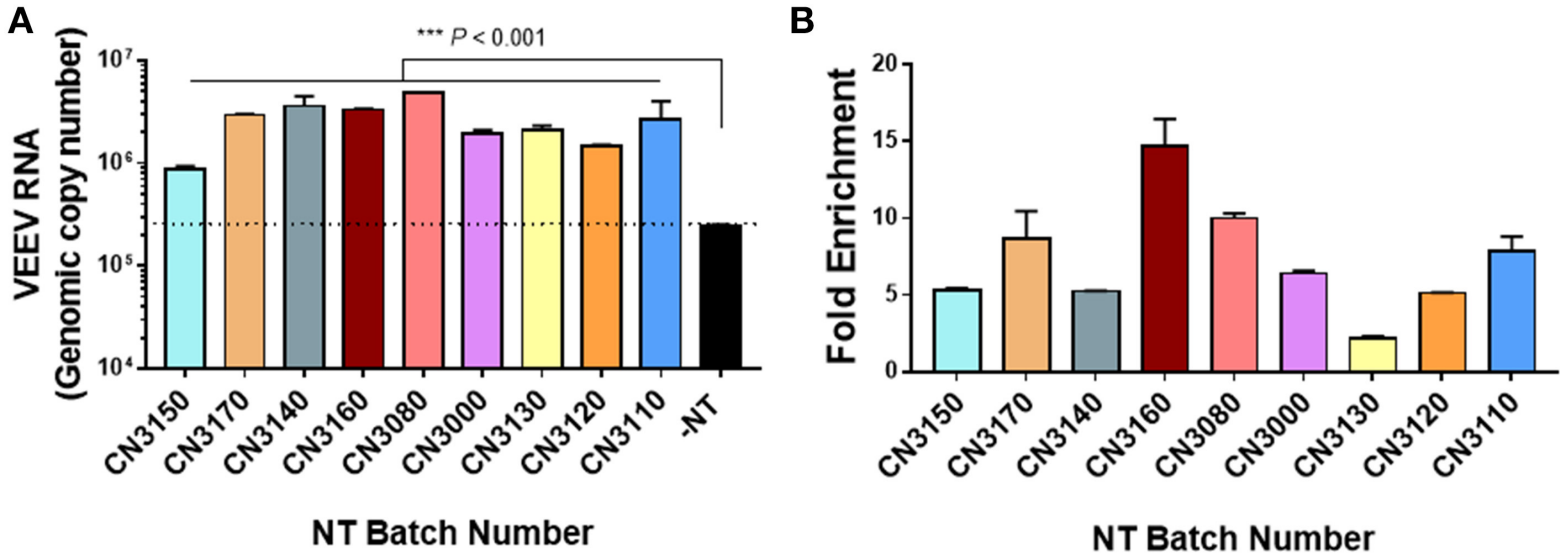
Screening of magnetic NT particles for the ability to enrich VEEV TC-83. VEEV at 1 × 106 PFU/mL was spiked into human blood followed by incubation with nine different magnetic NT particles (0.5 mg of NT/ml of sample). **(A)** The genomic copies of VEEV were determined by RT-qPCR after incubation for 30 min in the presence of NT particles. The dashed line indicates the minimal threshold for identification of enrichment. **(B)** Fold enrichment was calculated based on the following formula: NT-captured RNA/total input viral RNA. Data were presented as mean ± SEM from results of two independent experiments (*n* = 2). Statistical significance was determined by Kruskal-Wallis test where *P*-value under 0.05 was considered significant.

**Figure 2:**
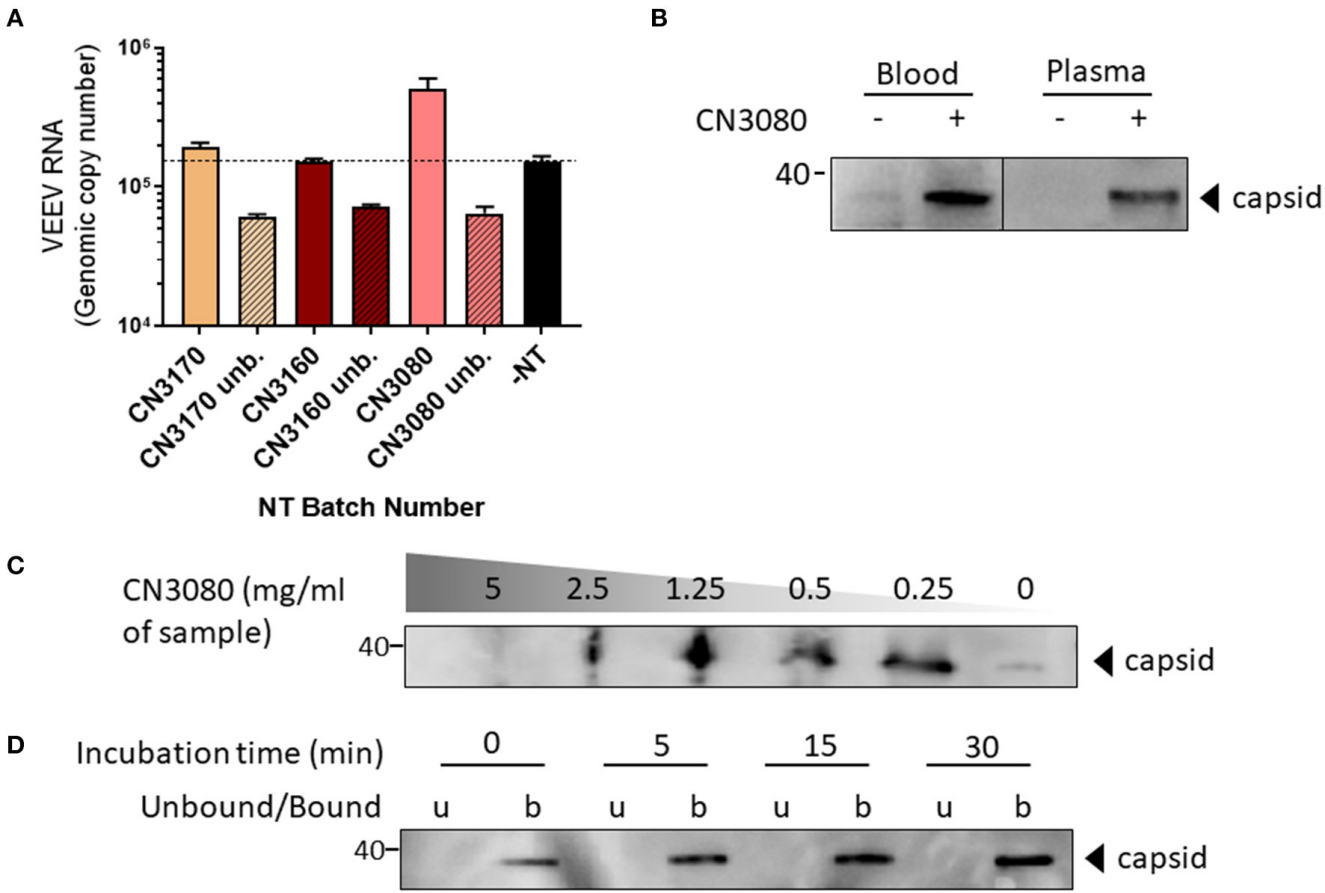
Validation of CN3080 for enrichment of VEEV. **(A)** VEEV at 1 × 10^6^ PFU/mL was spiked into human blood followed by incubation with CN3170, CN3160, or CN3080 magnetic NT particles (1.25 mg of NT/ml of sample). Unbound (Unb) = virus remaining in solution following NT particle incubation, –NT = samples processed without NT particles. The dashed line emphasizes the minimal threshold of enrichment. **(B)** VEEV TC-83 was spiked into blood or plasma at a final concentration 1 × 10^6^ PFU/mL and incubated with CN3080 (1.25 mg of NT/ml of sample). The level of VEEV capsid protein was determined via western blot analysis. **(C)** The concentration-dependent efficiency of CN3080 to preserve VEEV-TC83 was established using different concentration of NT beads to human blood (as indicated). The efficiency of VEEV capture was judged based on the capsid levels visualized by western blot analysis. **(D)** Time-dependent incubation of VEEV TC-83 in presence of CN3080 at room temperature. Data were evaluated based on western blot analysis of the capsid levels captured from a 1:20 blood:PBS mixture spiked with VEEV.

NT particles contain affinity baits that enable interactions with analytes through poorly defined characteristics. For example, triazine dyes such as cibacron blue and reactive red 120 bind to proteins through hydrophobic and electrostatic interactions (28). Therefore, the initial step of our study was to screen the panel of magnetic NT particles shown in Table 1, to evaluate their efficacies in enriching VEEV-TC83. NT particles were mixed with blood-spiked VEEV-TC83 (0.5 mg of NT/ml of sample) and incubated for 30 min prior to determination of viral RNA levels by RT-qPCR. We screened 9 different types of NT particles and found that all the NT particles were capable of enriching VEEV, but to different levels (Figures 1A,B). To further confirm and test the limit of capture efficiency of the NT particles, we selected three batches of NT particles shown with the best binding efficiencies and enrichment performance, CN3170, CN3160, and CN3080, and mixed NT particles with the blood-spiked VEEV-TC83 (1.25 mg of NT/ml of sample). All NT particles were capable of enriching VEEV when used at this concentration, but CN3080 showed the most significant enrichment (bound/unbound = ~10, Figure 2A), suggesting that the NT particle conjugated with reactive red 120 chemical bait could be the most suitable for the following experiments. We also compared the ability of CN3080 to a non-magnetic version (CN1030) and found that CN3080 was more effective at capturing VEEV (Supplemental Figure 1).

**Table 1:** Nanotrap particles tested.

**NT#**	**Description**
CN3150	Nanotrap ice blue
CN3170	Nanotrap neutral red
CN3140	Nanotrap blue—custom magnetic
CN3160	Nanotrap basic fuchsin
CN3080	Nanotrap reactive red 120
CN3000	Nanotrap purple
CN3130	Nanotrap yellow
CN3120	Nanotrap orange
CN3110	Nanotrap blue

To confirm virion capturing by CN3080, we conducted western blot analysis toward the VEEV-TC83 capsid protein. The capsid protein is only detected in samples that have been incubated with CN3080, but not in samples without NT particles regardless of whether virus was spiked into blood or plasma (Figure 2B). These data demonstrate the documented ability of NT particles to enrich viral analytes that are below the limit of detection (20). To characterize the performance of CN3080 we utilized various concentrations of CN3080 ranging from samples without NT particles to a 5 mg of NT/ml of sample to determine the optimal concentration for VEEV capsid enrichment and detection. To our surprise, the lower concentrations (up to 1.25 mg of NT/ml of sample) seemed to exhibit the most abundant enrichment, but not higher concentrations like 5 and 2.5 mg of NT/ml of sample (Figure 2C). Next, we tested for the optimal incubation time required for NT particle capture of the majority of virions in a sample. Thus, we measured the viral capsid concentration at different incubation time-points. As early as after 5 min of incubation, VEEV capsid protein was detected and by 15–30 min, the capture ability started to reach a saturation level (Figure 2D). These data show that CN3080 magnetic NT particles provide efficient capture of VEEV TC-83 virions over short time periods and the binding efficiency of CN3080 to viral samples can be optimized by adjusting both the incubation time and the NT particle concentration. Based on these data, we chose an incubation time of 30 min and a NT particle concentration of 1.25 mg/ml of sample for the remainder of our experiments to ensure maximal VEEV capture.

### VEEV Binds to the Outside of NT Particles

**Figure 3:**
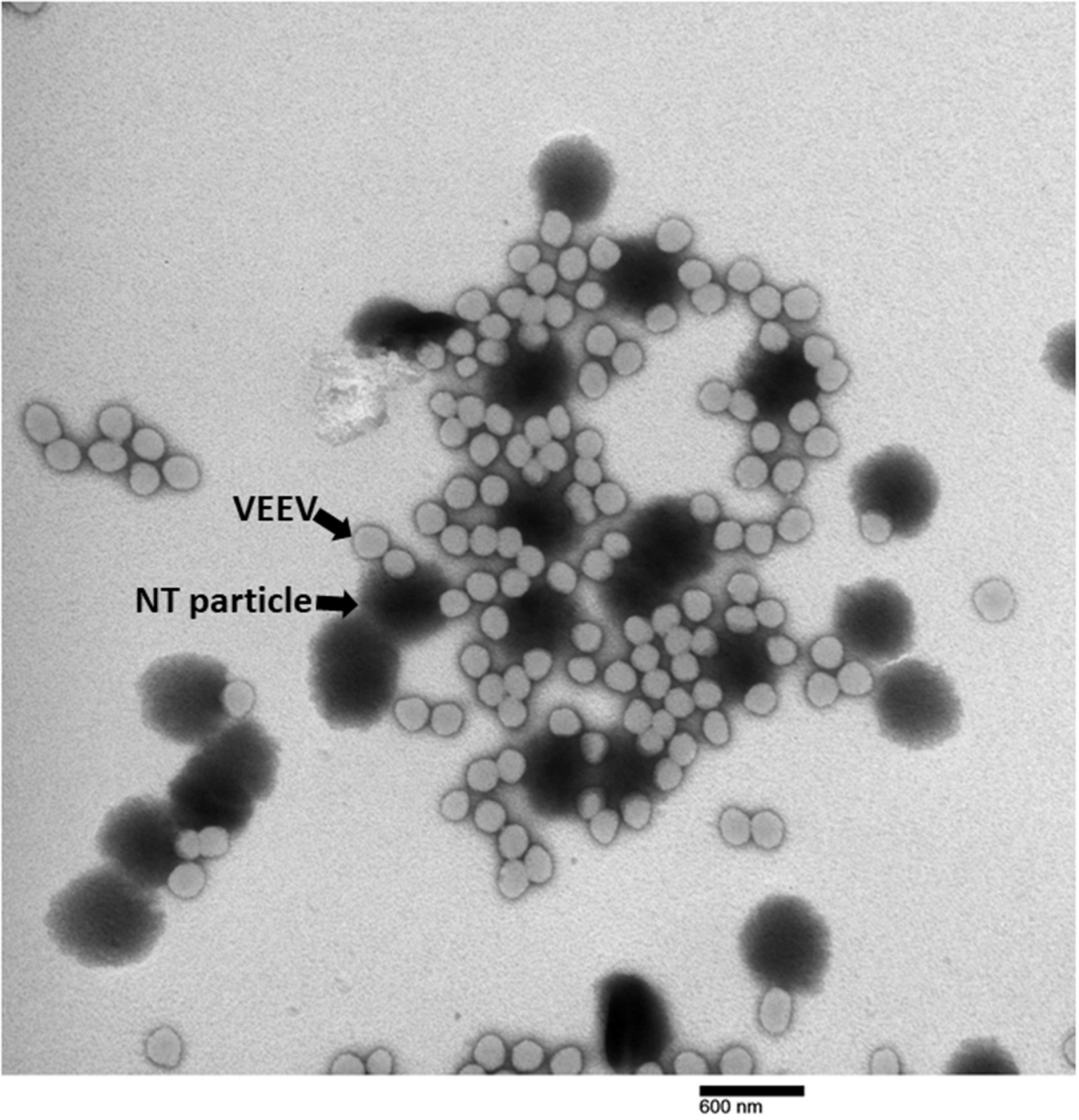
TEM of NT particle-captured VEEV virions. VEEV TC-83 spiked in DMEM was incubated with NT particles according to the standard protocol. TEM images were taken and magnification at 25,000X is displayed.

It has been shown in our previous studies that NT particles capture and enrich various viruses, such as influenza virus and RVFV (19, 21, 29), but it has never been shown if NT particles can directly bind to viruses. Using transmission electronic microscopy (TEM) we visualized the direct interaction between NT particles and virions. The photo in Figure 3 reveals that a single NT particle is capable of capturing multiple virions.

**Figure 4:**
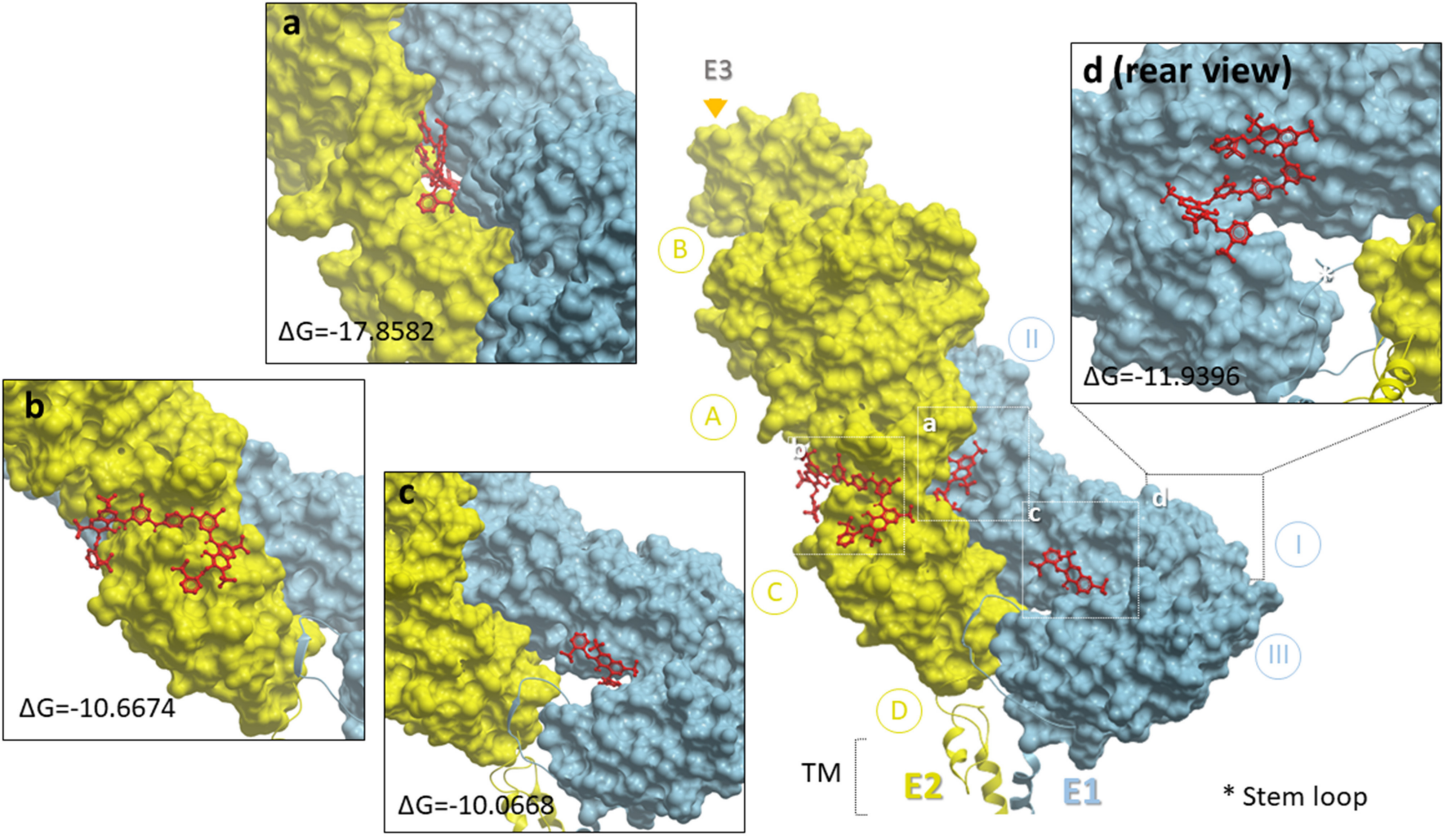
Docking simulation of reactive red 120 and heterodimer of VEEV E1 and E2 glycoproteins. The heterodimer of E1 (sky blue) and E2 (lemon chiffon) was depicted with MolBrowser v3.8 and subdomains of E1 or E2 were labeled as I, II, III (E1) and A, B, C (E2). The E1 and E2 subunits were alternatively presented in protein contact surface style in two distinct colors and the reactive red 120 in red was presented in ball-and-stick style. The star and triangle symbols indicated the locations of stem loop and E3 subunit, respectively, and the representative enlarged images were selected based on the lowest delta G free energy provided by Swissdock. TM, transmembrane domain. Inserted panel d displays one of the strong docking predictions which is located on the rear side of the E1 glycoprotein.

Our TEM data clearly showed that NT particles directly interact with viral particles. Therefore, we sought to elucidate the sites of the interaction between the NT particles and VEEV-TC83. We applied docking simulation via the SwissDock website and determined the free energy of binding (ΔG) toward the affinity bait, reactive red 120, and viral envelope proteins, E1 and E2 (PDB ID: 3J0C) (30). As shown in Figure 4, the docking predictions against the external domains of E1-E2 heterodimer protein reveal that the affinity bait overall interacted with E1 and E2 subunits where the strongest binding was predicted in the groove between E1 and E2 (panel 2a). The free energies of NT binding to each E1 and E2 subunits is summarized in Supplemental Table 1. In general, the prediction results suggest that there may be strong interactions between NT particles and VEEV envelope proteins which may potentially contribute to the viral enrichment exerted by CN3080.

### Stability of VEEV-TC83 Virions With or Without Nt Particles

**Figure 5:**
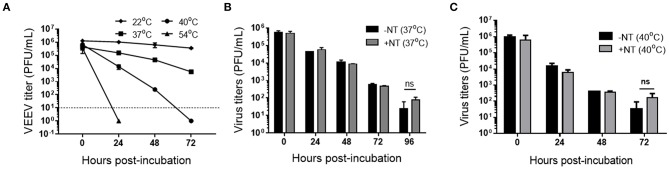
Detection of infectious VEEV TC-83 in human blood with and without CN3080. **(A)** VEEV was incubated at 22°C (room temperature), 37, 40, and 54°C for up to 72 h and viral titer determined by plaque assays. VEEV was diluted and spiked in human blood to 1 × 10^6^ PFU/mL and blood-spiked VEEV was co-incubated with CN3080 (1.25 mg of NT/ml of sample) or without CN3080 for 10 min at room temperature and infectious virus determined by plaque assays at 37°C **(B,C)** or 40°C for up to 96 h. Data were shown as mean ± SEM from results of at least two independent experiments (*n* = 2–4).

Previous studies have shown that NT particles are efficient in the preservation of RVFV and nucleoproteins in biologically relevant matrices (19, 29). However, the effect of NT in the stabilization of virions in a complex matrix such as whole human blood has never been tested before. Thus, after determination of the best NT composition that is most efficient in capturing VEEV TC-83, our next step was to test stability of the virus in whole blood at ambient and elevated temperatures for extended time periods. For this purpose, we spiked 10^6^ PFU of VEEV-TC83 in 0.5 mL of human whole blood and incubated samples at temperatures of 22, 37, 40, and 54°C for 0, 24, 48, and 72 h followed by measurement of viral infectivity by plaque assays. We observed a gradual drop of virus titers at 37 and 40°C, whereas viral infectivity remained relatively consistent at 22°C (Figure 5A). However, our several attempts to incubate virus at 54°C for 24 h repeatedly resulted in complete virus inactivation. It should be noted that at 54°C after 24 h of incubation the blood samples became solidified which made it impossible to completely resuspend samples in buffer for quantitation via plaque assay (Figure 5A). Therefore, we cannot exclude the possibility that some viral particles were captured by coagulated blood and still remained in the sample. As a result of these finding, for most of the following experiments we focus primarily on 37 and 40°C temperature conditions.

We next investigated whether CN3080 can prolong the stability of VEEV-TC83 in human whole blood. We found that the stability of VEEV at 37°C over the time points we tested was not substantially sustained in the presence or absence of CN3080 (Figure 5B) whereas the infectivity of VEEV TC-83 after 72 h of incubation at 40°C appeared to be better preserved despite the lack of statistical significance (Figure 5C). However, it should be noted that only once in four repeats did we observed two plaques developed by virus after 72 h of incubation without NT. In contrast, in all four repeats we were able to detect virus-produced plaques (ranging from 2 to 6 plaques per well) if samples were incubated in presence of CN3080. This indicates that the addition of CN3080 in samples may stabilize the VEEV virions and preserve infectivity for diagnostic purposes.

### NT Particles Help to Preserve Viral RNA and Capsid Protein Detection at Elevated Temperatures

**Figure 6:**
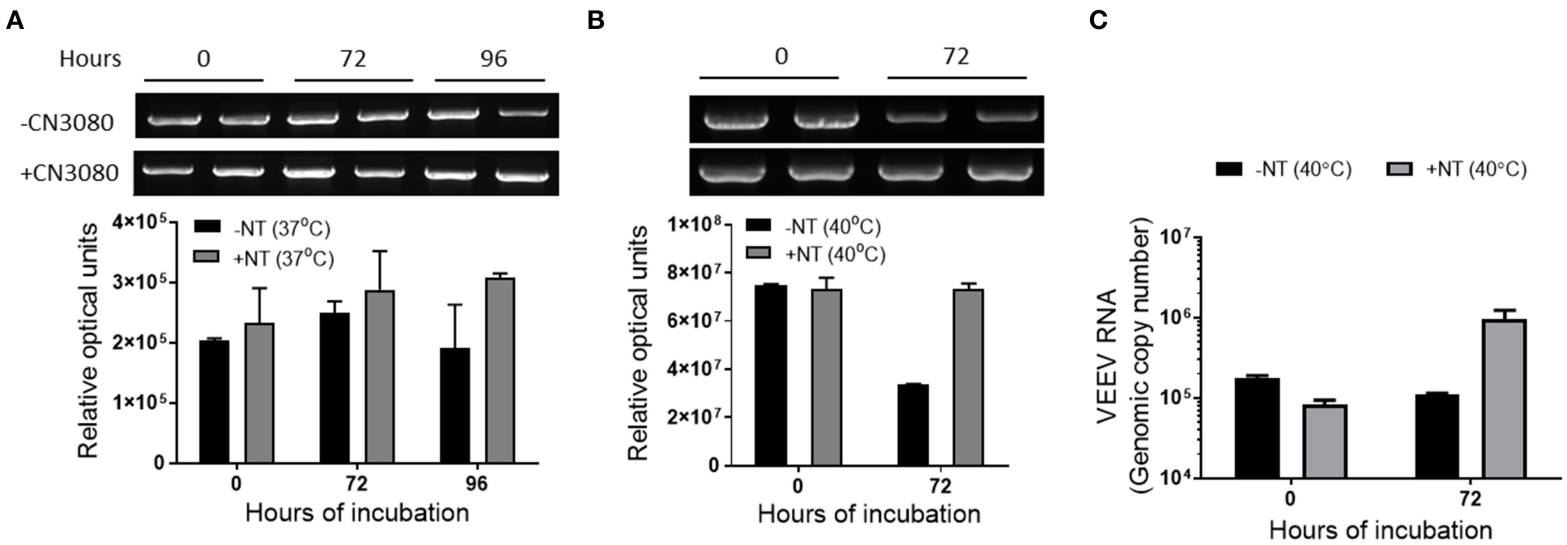
CN3080 efficiently preserved viral RNA in human blood. VEEV TC-83 was spiked in human blood to 1 × 10^6^ PFU/mL in the presence (1.25 mg of NT/ml of sample) or absence of CN3080 and incubated up to 96 h at different temperatures. The preservation of the VEEV TC-83 RNA at **(A)** 37°C and **(B)** 40°C was quantified and analyzed based on the densitometry analysis of the 1.5 kbp RT-PCR product of the VEEV genome. **(C)** Samples incubated at 40°C were also analyzed by RT-qPCR. Data plotted as mean ± SEM from two independent experiments.

The primary approach for confirming VEEV infection in suspected clinical cases is based on detection of the virus specific IgM and neutralizing antibodies. Reactions based on amplification of the nucleic acid is often used in fatal cases to confirm pathogen presence (5, 19, 31). RT-qPCR is a very reliable method and highly sensitive for the detection of minimal amounts of viral nucleic acids in the patient samples (11). However, this method is based on the detection of relatively short fragments of the viral genome and analysis of a longer stretch of viral RNA (vRNA) would provide a more stringent measure of RNA stability. Thus, we investigated the stability of both long viral RNA (1.5 kb product) by RT-PCR and short vRNA (75 bp) by RT-qPCR. VEEV was spiked in blood and incubated in the presence or absence of CN3080 for the indicated amount of time, followed by RNA purification, cDNA synthesis and PCR or qPCR reactions. Minimal differences in long vRNA levels were observed in samples incubated at 37°C (Figure 6A). CN3080 overall stabilized long vRNA and increased its detection by RT-PCR especially at higher temperature (40°C) conditions compared to samples that were incubated without NT particles (Figure 6B). Densitometric analysis of the PCR products indicated preservation of long vRNA in the presence of CN3080 after 72 h of incubation at 40°C. Likewise, RT-qPCR results indicated preservation of short vRNA after 72 h at 40°C (Figure 6C).

**Figure 7:**
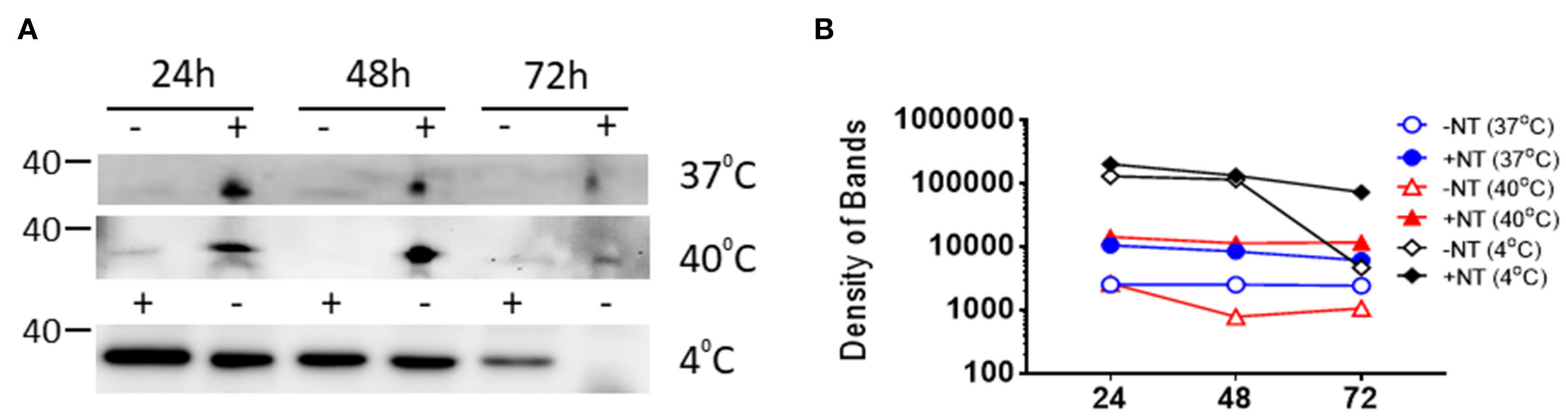
CN3080 efficiently preserved VEEV capsid protein in human blood. **(A)** 2.25 × 10^8^ PFU/mL of VEEV TC-83 was added to blood diluted 1:20 in PBS followed by incubating in the presence (1.25 mg of NT/ml of sample) or absence of CN3080 to assess the preservation efficacy of NT against VEEV capsid protein degradation over 0, 24, 48, and 72 h at 4, 37, and 40°C. **(B)** Densitometry analysis of western blot images shown in **(A)**.

For comparison, we also monitored the degree of VEEV capsid protein decay in the presence or absence of CN3080. Given the difficulty in analysis of blood samples via western blot, these experiments were performed in blood diluted 1:20 with PBS. As a starting point we tested the ability of CN3080 to prevent capsid protein degradation at standard blood storage conditions. For this purpose, we incubated virus with and without CN3080 at 4°C and assessed by western-blot analysis the amount of capsid protein in the incubated samples. Even though VEEV capsid was overall more stable at 4°C, we observed a decrease in capsid protein detection by 72 h, which was rescued by incubation with CN3080 (Figure 7A, bottom panel). Western blot analysis of the samples incubated at 37 and 40°C also demonstrated that only when incubating with CN3080, capsid can be preserved and more available for detection (Figures 7A,B). Thus, NT particles not only profoundly enriched capsid protein in all samples at all temperature conditions but in their presence the capsid protein degradation was significantly reduced at high temperature. Results from the above RT-PCR and protein analyses suggest that CN3080 could efficiently enrich and preserve VEEV RNA and capsid protein against the harsh temperature storage conditions.

### Stability of Viral Titer of VEEV-TC83 With or Without NT Particles at Various Humidity Conditions

**Figure 8:**
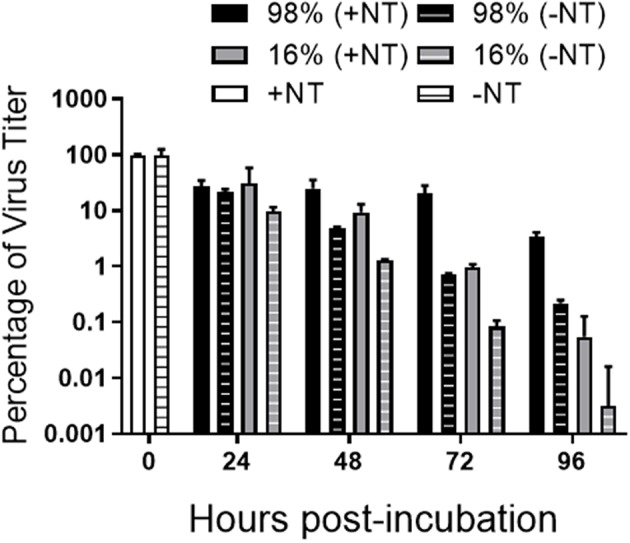
CN3080 preserves infectious VEEV in humid and dry environments. 2.25 × 10^8^ PFU/mL of VEEV TC-83 was added to blood diluted 1:20 in PBS followed by incubating with (1.25 mg of NT/ml of sample) or without CN3080 at 37°C in 16 or 98% humidity conditions and preservation was measured by plaque assays with CN3080 or without CN3080. Values below the detection threshold were set to 1. Data are plotted as the geometric mean ± SEM from results of two independent experiments (*n* = 2).

Some studies have shown that relative humidity can affect the stability of airborne viruses including aerosolized VEEV (32–34). Therefore, we investigated whether CN3080 could contribute to the blood-spiked VEEV stability across environmental humidity ranges. We placed the viral samples with or without CN3080 in two incubators with relative humidity at 16 or 98% at 37°C and incubated them for up to 96 h followed by determination of viral titers. Our plaque assay results indicated that viral titers of the blood-spiked VEEV-TC83 were preserved at both 16% and 98% humidity when incubating with CN3080 for 72 and 96 h post-incubation (Figure 8). Samples incubated in higher humidity conditions were generally more stable, but the addition of CN3080 further enhanced the stability regardless of the level of humidity. Our data support the conclusion that NT particles efficiently stabilize infectious virus at elevated and dry humidity conditions for an extended amount of time.

### NT Particles Stabilized VEEV Capsid Protein in Blood Samples Collected From VEEV-TC83 Infected Mice

**Figure 9:**
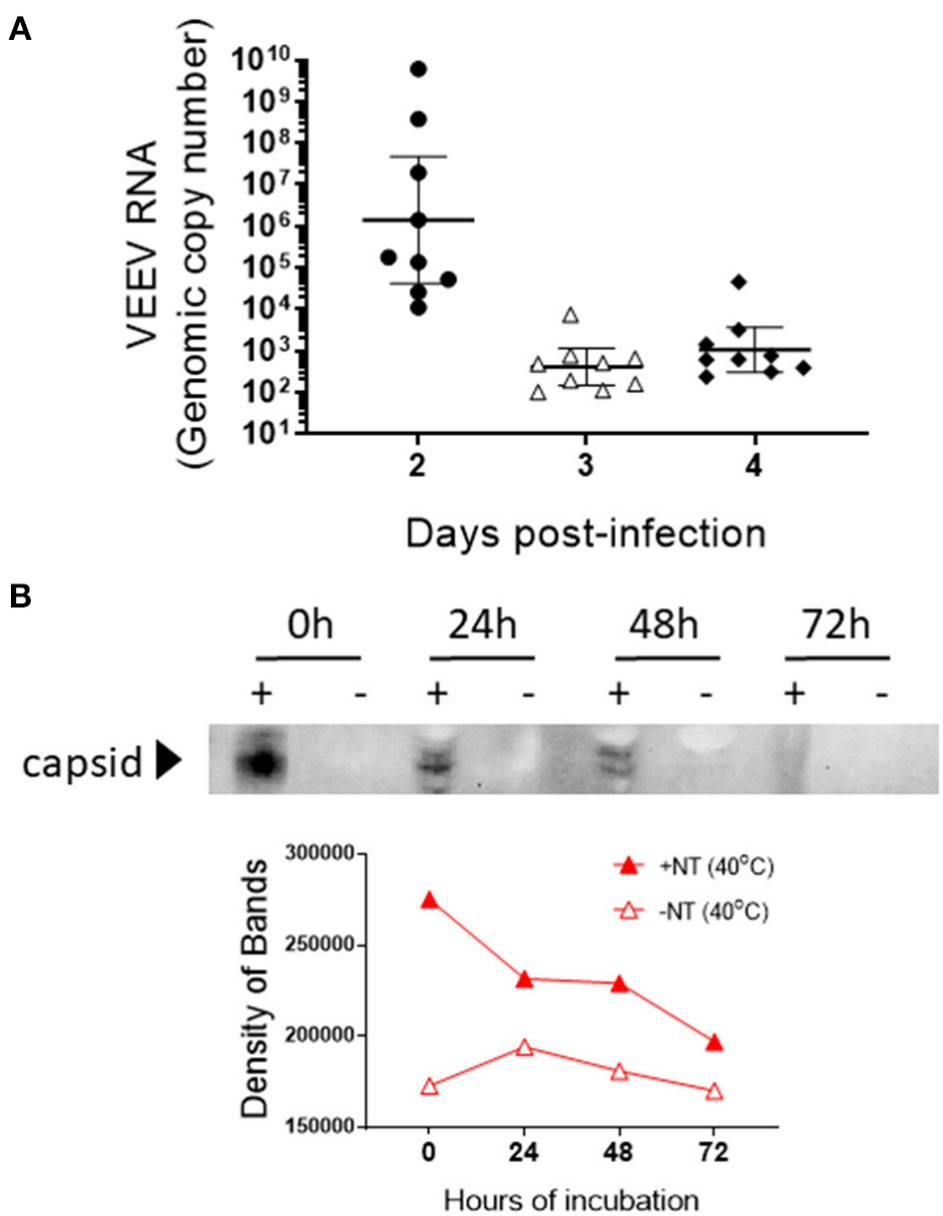
CN3080 preserves and enhances the detectable level of VEEV-TC83 capsid protein in blood of infected mice. **(A)** Mouse blood was collected at day 2, 3, and 4 after intranasal infection of C3H/H3N mice (*n* = 9 at each time point) with VEEV-TC83. The concentration of VEEV RNA in mouse blood was determined by RT-qPCR. Data are plotted as the geometric mean ± SEM. **(B)** Blood collected at day 2 was incubated with (1.25 mg of NT/ml of sample) or without CN3080 for 72 h at 40°C. Viral capsid protein preservation was evaluated by western blot analysis. The bottom panel displays the densitometry analysis of the western blot results.

To better illustrate the application of CN3080 in clinical samples, we infected C3H/HeN mice via intranasal infection of VEEV-TC83 and measured the viral load in mouse blood at 2, 3, and 4 days post-infection (d.p.i.) followed by supplying CN3080 to demonstrate the preservation capability of NT particles *ex vivo*. Since the mouse blood samples from day 2 showed the highest viral RNA concentration among all tested time points (Figure 9A), we used blood harvested from mice at 2 d.p.i., added CN3080, and compared the capsid protein stability with or without NT particles at 40°C for the indicated amount of time. This experiment was performed with whole blood. In the absence of NT particles, VEEV capsid was undetectable at all time points (Figure 9B). Incubation of mouse blood with CN3080 enable capsid detection at each time point, highlighting the potential use of NT particles in clinical samples.

## Discussion

NT particles are made of a hydrogel matrix and can be customized with various affinity baits such as acrylic acid, Cibacron blue dye, and reactive red dye by polymerization (35). As such, they can bind a range of biomolecules. In our previous studies, we have found that NT particles containing a reactive red 120 affinity bait allows NT particles to capture a wide range of RNA viruses (19, 29). Using the newly developed magnetic NT particles could simplify workflows allowing the isolation of NT particles in 3–5 s instead of long centrifugation steps. This processing allows the virions present in clinical samples and bound to NT particles to be easily and quickly separated from body fluids using either manual or automated methods. However, one of the obstacles in pathogen identification in whole blood is the natural abundance of host proteins and red blood cells that prevent direct identification of the pathogen from blood and often require additional preparation steps. Another inconvenience related to working with whole blood or other body fluid sample is a necessity to keep it at low temperatures during transportation. Such obstacles make blood a very difficult matrix for sample preparation and diagnostic analysis. However, despite the existence of the above-mentioned obstacles, we observed efficient separation of VEEV viral particles from blood by NT particles at various temperature and humidity conditions. Moreover, NT particles efficiently preserved infectious VEEV TC-83 at conditions where it was otherwise undetectable.

As much as our NT particle work has shown a significant benefit in preservation and enhancing detection of VEEV virions confirmed by stabilization of viral capsid protein, viral RNA, as well as infectious virions, there are limitations in the use of NT particles. When the environmental conditions are too harsh, the NT particles are only capable of partially alleviating the negative effects. Despite the trend showing more preservative via NT particles, not all viral titers at the later time points exhibited statistical differences, suggesting the preservation of NT particles may not be sufficient to prevent the ultimate decay of virions under the most extreme environmental conditions. Another limitation for the use of NT particles is the difficulty in separating virions from NT particles. We have previously shown very strong binding of RVFV virions to NT particles (19). Working with RVFV we were able to elute only 5.5% from the total virions bound to NT. For this purpose we used sodium chloride in the highest concentration that does not induce virus degradation (2 M), but even this concentration was not enough to completely elute virions from NT. Although strong lysis buffers, such as Trizol reagent and RIPA buffer, can be utilized for precipitating RNA or extracting viral proteins for diagnosis purposes, we have not found a method to elute the virions while retaining the viral infectivity. As shown in our electronic micrograph (Figure 3), a NT particle is potentially bound with dozens of virions, indicating the efficient capturing ability of NT particles. However, the principle of plaque assay is to dilute viral samples until a single virion can form a plaque, so called plaque-forming unit, visualized, and countable by investigators. Overwhelming capture affinity of NT particles to virions is an obstacle for the accuracy of the plaque assay method, which might lead to an underestimation of initial viral titers (Figure 5). Therefore, a better way to elute virions needs to be developed to enable more accurate viral titer determination. One possibility for future testing is to competitively elute VEEV via addition of increasing concentration of the reactive red affinity bait.

Despite the limitations discussed above, our data indicate that NT particles are a useful innovation to researchers. The NT particles can be customized with various affinity baits to bind different microorganisms or biomolecules. In addition to the viruses, NT particles have been shown to be capable of binding the outer surface protein of *Borrelia*, the bacteria that causes Lyme disease, and increasing the specificity of diagnosis for early stage of Lyme disease (18). Also, biomarkers in clinical fluid samples or secreted extracellular organelles like exosomes can be captured and the sensitivity of their detection enhanced via NT particles (36–38). The ability of the NT particles to preserve VEEV and its components including capsid protein and genomic RNA in such complex matrix such as whole blood highlight an importance to further study the capabilities of the NT particles.

Overall, the magnetic NT particle presented in this study demonstrated efficient binding affinity to capture one of the biological warfare agents, VEEV, allowing laboratory technicians to detect this particular pathogen from clinical samples of patients or soldiers in the front line. Moreover, the high efficiency in preservation of live pathogen and its proteins and nucleic acids make NT particles an attractive candidate to be used as a stabilization agent in a new blood collection medical device that are currently undergoing development and testing. Hence, utilization of the NT particles can be an alternative method to enhance the surveillance system to monitor and prevent the outbreaks. Our study provides mechanistic insights for broad use of NT particles.

## Data Availability Statement

All datasets generated for this study are included in the article/Supplementary Material.

## Ethics Statement

The animal study was reviewed and approved by George Mason University Institutional Animal Care and Use Committee.

## Author Contributions

IA, S-CL, AP, LA, TM, BL, MH, and KK-H designed experiments. IA, S-CL, and CL performed the experiments and analyzed the data. MS provided TEM data and analysis. IA and S-CL wrote the manuscript. AP, MS, LA, BL, MH and KK-H edited the manuscript.

### Conflict of Interest

AP and BL are employed by the company Ceres Nanosciences, Inc. KK-H is a member of the Scientific Advisory board at Ceres Nanosciences, Inc. BL is a shareholder at Ceres Nanosciences, Inc. The nanoparticles used in this study were research grade and provided by Ceres Nanosciences, Inc. which are not commercially available products. The authors declare that Tasso Inc had no role in the design of the study and collection, analysis, interpretation of the data or writing of the manuscript. The remaining authors declare that the research was conducted in the absence of any commercial or financial relationships that could be construed as a potential conflict of interest.
