# Supplementary material for: Magnetic Nanotrap Particles Preserve the Stability of Venezuelan Equine Encephalitis Virus in Blood for Laboratory Detection
Source: Front Vet Sci. 2020 Jan 28;6:509. doi: 10.3389/fvets.2019.00509 (PMC6999085; doi:10.3389/fvets.2019.00509)
Supplement: Supplementary file 1 [file Presentation_1.PPTX]

## Slide 1
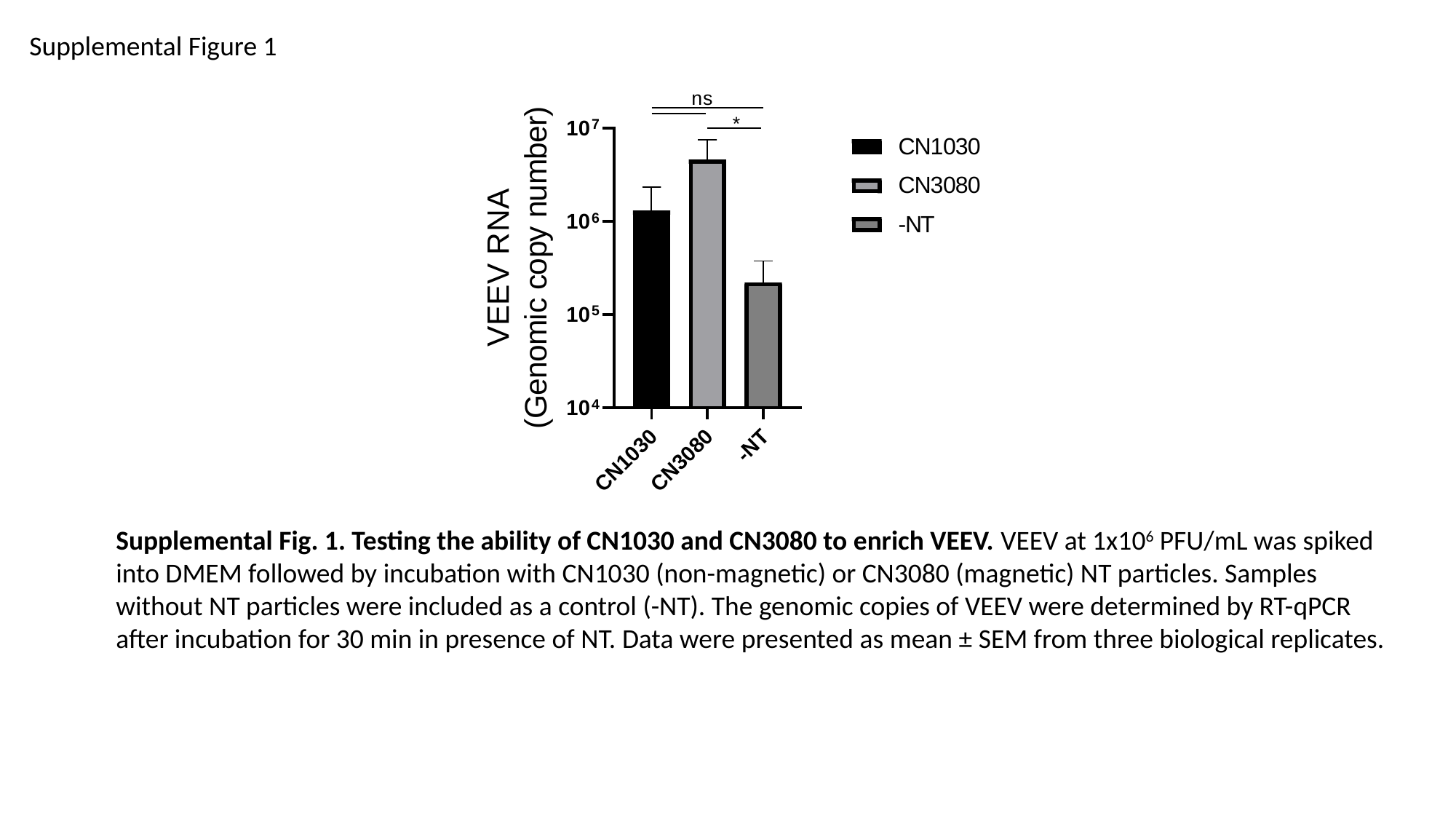

Supplemental Figure 1
Supplemental Fig. 1. Testing the ability of CN1030 and CN3080 to enrich VEEV. VEEV at 1x10^6^ PFU/mL was spiked into DMEM followed by incubation with CN1030 (non-magnetic) or CN3080 (magnetic) NT particles. Samples without NT particles were included as a control (-NT). The genomic copies of VEEV were determined by RT-qPCR after incubation for 30 min in presence of NT. Data were presented as mean ± SEM from three biological replicates.
